# Altered Brain Functional Hubs and Connectivity Underlie Persistent Somatoform Pain Disorder

**DOI:** 10.3389/fnins.2019.00415

**Published:** 2019-04-30

**Authors:** Qu Liu, Xian-chun Zeng, Xiao-Mei Jiang, Zhen-hua Zhou, Xiao-fei Hu

**Affiliations:** ^1^Department of Neurology, Southwest Hospital, Third Military Medical University (Army Medical University), Chongqing, China; ^2^Department of Radiology, Guizhou Provincial People’s Hospital, Guiyang, China; ^3^Department of Centre for Disease Prevention and Control, Chengdu Military Region, Chengdu, China; ^4^Department of Radiology, Southwest Hospital, Third Military Medical University (Army Medical University), Chongqing, China

**Keywords:** persistent somatoform pain disorder, emotional disturbance, resting-state functional MRI, degree centrality, Granger causality analysis

## Abstract

This study investigated the degree of brain functional impairment in persistent somatoform pain disorder (PSPD) by examining changes in the patterns of brain functional hubs. Resting-state functional magnetic resonance imaging was performed in 21 PSPD patients with headache as the main symptom and 17 sex- and age-matched healthy controls. Degree centrality (DC) analysis as well as the connectivity among these hubs by functional connectivity (FC) analysis and Granger causality analysis (GCA) were performed to characterize abnormal brain networks in PSPD (Gaussian random field corrected: *P* < 0.001, *Z* > 3.09). The relationships between DC and connectivity and clinical parameters were also examined. DC values in the bilateral inferior occipital gyrus (IOG), bilateral calcarine fissure (CAL), and left paracentral lobule (PCL) and FC values of right IOG–left CAL, right IOG–right CAL, right IOG–left IOG, left CAL–right CAL, left CAL–left IOG, left CAL–left PCL, right CAL–left PCL, and left IOG–left PCL were lower in PSPD patients as compared to controls. A negative causal effect from the left CAL to the left paracentral lobule and a positive effect from the right CAL to the right IOG were observed in PSPD patients. Abnormal DC, FC, and signed-path coefficients in PSPD patients were negatively correlated with self-rating anxiety and depression scale scores. These results indicate that altered functional hubs and connectivity patterns in the somatosensory cortex may reflect emotional disturbance in PSPD patients.

## Introduction

Persistent somatoform pain disorder (PSPD) is characterized by medically unexplained somatic symptoms (Smith et al., 2000). PSPD patients experience long-term, persistent, severe, and distressing pain (typically lasting for over 6 months) that cannot be attributed to a specific cause (World Health Organization [WHO], 1993; American Psychiatric Association [APA], 2013). Various studies have described emotional and cognitive impairment in PSPD patients (Luo et al., 2014, 2016). For instance, alexithymia and anxiety are very common symptoms in adolescents with PSPD (Burba et al., 2006). Others have reported that PSPD patients exhibited deficits in emotion perception but normal ability to recognize facial expressions (Schonenberg et al., 2014). Additionally, lower scores on the quality of life sub-scale of the 36-Item Short Form Survey were shown to be correlated with pain, depression, and anxiety scores in PSPD patients (Luo et al., 2014). However, none of these studies investigated the mechanism(s) underlying the impairment of brain function in PSPD patients.

Functional magnetic resonance imaging (fMRI) provides an effective means for studying PDSD-related cortical alterations. A task-based fMRI study showed that the prefrontal–temporal–limbic circuit was activated in PSPD patients in response to pinprick pain stimulation under negative emotional conditions, suggesting a potential role for emotion in pain processing and the pathophysiology of PSPD (Luo et al., 2016). Meanwhile, a resting-state (rs-)fMRI study reported changes in regional homogeneity (ReHo) in brain regions related to pain in PSPD patients during the resting state, including the default mode network, somatosensory and prefrontal cortices, and posterior cerebellum (Huang et al., 2016). A functional connectivity analysis showed altered co-activation within aberrant resting-state networks such as sensorimotor, default-mode, and salience networks, suggesting that PSPD patients experience large-scale reorganization (Zhao et al., 2017). However, these studies have focused on local spontaneous brain activity within selected brain regions based on a priori assumptions or by independent components analysis, and have not fully characterized the functional connectome of PSPD patient brains.

Degree centrality (DC) is a measure of network organization based on graph theory that has been applied to the identification of candidate functional hubs in several rsfMRI studies (Cole et al., 2010; Fransson et al., 2011; Di et al., 2013). This has allowed characterization of the functional relationships of a given node (voxel) within the full-brain connectivity matrix as opposed to relationships with specific nodes or networks (Tomasi and Volkow, 2011; Zuo et al., 2012). Thus, DC can reveal the complexity of the functional connectome of PSPD patients.

After representing the hubs of the brain functional connectome using DC, the subsequent analysis of the complexity and patterns the interactions among these functional hubs should been investigated with undirected and directed functional connectivity (FC). Directed FC can be estimated with traditional FC (Biswal et al., 1995), and Granger causality model could investigate positive and negative signed-path coefficients in an directed manner (Seth et al., 2015). Combining traditional FC and Granger causality analysis (GCA) get a more complete characterization of connectivity patterns among brain functional hubs in PSPD.

In this study, we tested the hypothesis that aberrations in brain functional hubs and their connectivity contribute to PSPD. Candidate functional hubs were identified based on DC, and FC analysis and GCA were used to investigate their interconnectivity. We also investigated the relationships among changes in brain function, clinical data, and neuropsychological performance. Our results provide insight into the neurological basis for the impairment of brain function in PSPD.

## Materials and Methods

### Participants

This study was approved by the Ethics Committee of Southwest Hospital (Chongqing, China), and all study subjects provided written, informed consent to participate in the study.

Consecutive PSPD patients were recruited from inpatients at the hospital and healthy controls (HCs) were recruited from the community between December 2016 and December 2017. A total of 21 patients were diagnosed with PSPD by an experienced specialist according to ICD-10 criteria; inclusion criteria for this group were as follows: (1) right-hand dominance (which was tested with the Edinburgh Handedness Inventory); (2) age between 18 and 65 years; and (3) clinical pain (headache as the main symptom) persistently for at least 6 months. All patients’ treatment and all the medication like antidepressants and pain-relieve drugs were taken after MRI scan. Additionally, 17 HCs matched in terms of age, sex, and handedness were recruited.

Exclusion criteria for all subjects were as follows: (1) presence of pain symptoms due to severe somatic disease; (2) pre-existing neurological or psychiatric disorder (including a history of seizures, global cognitive impairment, aphasia, neglect, substantial sensory disturbance, severe “major depressive disorders” in Statistical Manual for Mental Disorders (DSM)-V or claustrophobia); (3) existence of uncontrolled disease such as congestive heart failure, hypertension, cerebrovascular disease, and thyropathy; (4) metal clips in the brain; or (5) pneumonia at the time of enrollment.

### Clinical Assessment

The Visual Analog Scale (VAS), Zung Self-Rating Anxiety Scale (SAS), and Zung Self-Rating Depression Scale (SDS) were used to assess pain characteristics, anxiety symptoms, and depression symptoms in each patient, respectively.

The VAS is a psychometric response scale that is the most widely used quantitative approach for evaluating pain (Reips and Funke, 2008). Clinical pain was scored from 0 (no pain) to 10 (extreme pain).

The SAS is a 20-item self-assessment devised to measure anxiety levels based on scores for cognitive, autonomic, motor, and central nervous system symptoms (Zung, 1971, 1974), with total raw scores ranging from 20 to 80. The raw score is then converted to an anxiety index (normal, 20–44; mild to moderate, 45–59; marked to severe, 60–74; and extreme, 75–80) for clinical interpretation of anxiety level.

The SDS is a self-administered survey for assessing the level of depression symptom (Zung et al., 1965), with scores ranging from 20 to 80. Depression scores were categorized as follows: normal range, 20–44; mildly depressed, 45–59; moderately depressed, 60–69; and severely depressed, ≥70.

### MRI Data Acquisition

The MRI scan was performed on the same day that clinical data were obtained. Images were acquired using a 3.0 T Siemens Tim Trio whole-body MRI system (Siemens Medical Solutions, Erlangen, Germany). Subjects were instructed to stay awake and close their eyes, and to try not to think of anything. Foam padding and earplugs were used to reduce head motion and scanner noise. Imaging data were collected transversely using an echo-planar imaging sequence with the following settings: TR = 2000 ms, TE = 30 ms, flip angle = 90°, FOV = 192 mm × 192 mm, in-plane matrix = 64 × 64, thickness = 3 mm, voxel size = 3.0 mm × 3.0 mm × 3.0 mm. For each subject, a total of 240 volumes were acquired with a scan time of 480 s. T1-weighted structural images were collected by volumetric three-dimensional magnetization prepared with the following rapid-acquisition gradient-echo sequence (TR = 1900 ms, TE = 2.52 ms, flip angle = 9°, slice thickness = 1 mm, slices number = 176, FOV = 256 mm × 256 mm, matrix size = 256 × 256 and voxel size = 1 mm × 1 mm × 1 mm and sagittal scanning).

### MRI Data Analysis

All preprocessing steps were performed using the Data Processing Assistant for Resting-State fMRI (DPARSF2.3^1^), which is based on the Statistical Parametric Mapping (SPM8) program^2^ (Chao-Gan and Yu-Feng, 2010). Intracranial tissue segmentation was performed with Voxel Based Morphometry Toolbox 8 v.435 software. The main steps included individual 3D T1-weighted structural images co-registered to the mean of the realigned EPI images, and segmentation of intracranial tissue into gray matter, white matter, and cerebrospinal fluid (CSF), which automatically produced volume information for each brain tissue. Gray matter was smoothed with a full-width at half-maximum of 4 mm.

Prior to preprocessing of functional data, (1) the first 10 volumes were removed, (2) slice timing was performed, (3) re-alignment was performed to correct head motion. Based on the recorded motion correction estimates, the subjects with more than 2.0 mm in any direction (x, y, or z) or more than 2.0° at any angle were excluded from the study. The Friston 24-parameter model was used to regress out head motion effects (Yan et al., 2013). Other nuisance variables including white matter signal and CSF were regressed out. (4) Individual functional images were normalized in Montreal Neurological Institute (MNI) space for inter-subject comparison. (5) The resultant images were smoothed with a full-width at half-maximum of 4 mm. (6) De-trending was performed. (7) Data were bandpass filtered (0.01–0.08 Hz).

Based on the pre-processing, DC calculations were performed using DPARSF in a voxel-wise manner (Beucke et al., 2013; Zhou et al., 2016). First, the preprocessed functional data sets were subjected to voxel-based whole-brain correlation analysis. The time course of each voxel within the gray matter mask from each participant was correlated with the time course of every other voxel, which generated a correlation matrix. An undirected adjacency matrix was then obtained by thresholding each correlation at *r* > 0.25 (Beucke et al., 2013). Then, the DC was computed as the sum of the weights of the significant correlations for each voxel. Finally, by subtracting the mean DC across the entire brain and then dividing by the standard deviation of the whole-brain DC, these individual-level voxel-wise DC maps were standardized into a *z*-score. A smoothing kernel of 4 mm was applied. Peak MNI coordinates of candidate brain functional hubs identified by DC inter-group analyses and were used as seeds in subsequent analyses, and a sphere ROI was generated for each of the brain sites, the center of which corresponded to the peak voxel with a 6-mm radius. An independent samples *t*-test of DC maps between groups were performed by REST v.1.8 software^3^, with age and sex as covariates. Multiple comparisons correction was performed using a Gaussian random field (GRF) at *P* < 0.001, *Z* > 3.09 (Hayasaka et al., 2004).

Connectivity among the ROIs was analyzed using REST v.1.8 software, including FC and GCA analyses. For FC, correlation analysis of time course was performed between every two of the seed region for each subject. Fisher’s r-to-z transformation was applied to improve the normality of the FC maps. For GCA, signed-path coefficients between ROIs were computed in multivariate mode for subsequent parametric statistical analyses (Hamilton et al., 2011). The GCA was performed on the gray matter mask using REST tool-box (see text footnote 1). First, we used Granger approach that examined the time lagged effects between two nodes to infer the causal effects between regions (Chen et al., 2011). The signed-path coefficient generated using a time lag order of 1 TR (2 s) was used to estimate the probable excitatory or inhibitory effects of the directed physiological influence (Hamilton et al., 2011). The bivariate GCA accounted for the physiological probability of simultaneously bidirectional influences in the brain (Palaniyappan et al., 2013). In addition, the path coefficients were normally distributed and could be used in parametric statistical analysis for group level inference (Hamilton et al., 2011). Differences in FC and GCA between PSPD patients and HCs were computed with independent samples *t*-test using SPSS software (SPSS Inc., Chicago, IL, United States), with age and sex as covariates. Multiple comparisons correction was also performed using multiple comparisons by Bonferroni correction (*P* < 0.05/10 for FC and *P* < 0.05/20 for GCA).

### Correlation Analysis

The relationships among DC, connectivity, clinical parameters, and neuropsychological test scores were examined by partial correlation analyses using SPSS software in all study participants while controlling for the effects of covariates used in the inter-group analyses of DC maps. *P* < 0.05 was considered statistically significant.

## Results

### Comparison of Demographic and Clinical Data

There were no significant differences between groups in terms of age, sex, and education. PSPD patients had higher SAS and SDS scores (all *P* < 0.001) (Table 1).

**TABLE 1:** Demographic characteristics and clinical assessment of participants.

	PSPD (*n* = 21)	HCs (*n* = 17)	t/χ^2^-value/	*p*-Value
Age (years)	42.9 ± 8.5	43.3 ± 10.7	0.143^#^	0.887
Sex (male: female)	4/17	3/14	0.12*	0.912
Duration of illness (years)	3.5 ± 2.57	–	–	–
VAS	5.69 ± 2.10	–	–	–
SAS	67.3 ± 10.0	41.1 ± 10.8	7.379^#^	<0.001
SDS	68.6 ± 6.5	44.8 ± 9.1	9.288^#^	<0.001

*χ^2^ test. ^#^Independent *t*-test. VAS, Visual Analog Scale; SAS, Self-Rating Anxiety Scale; SDS, Self-Rating Depression Scale.

### DC Analysis

**FIGURE 1:**
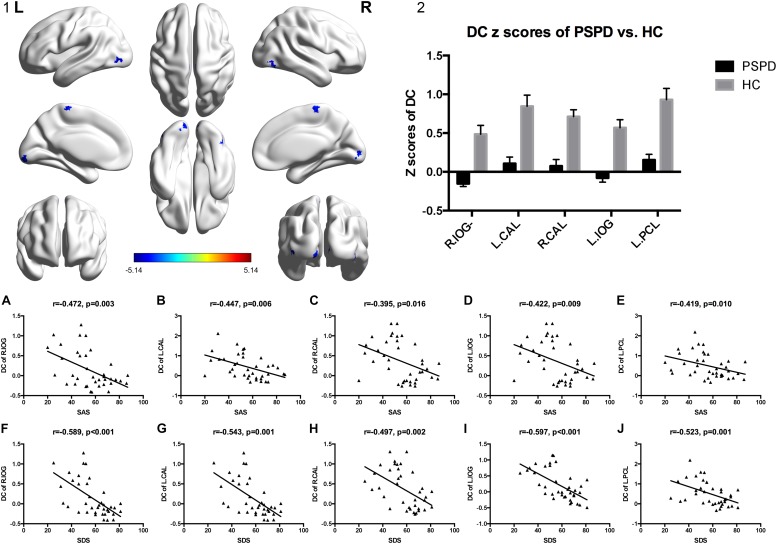
DC value distribution of inter-group comparisons and correlations among the altered DC and PSPD-related parameters (SAS and SDS). **(1)** The significantly altered DC map in the PSPD group. **(2)** Comparison of DC value between the two groups (GRF corrected: *P* < 0.001, *Z* > 3.09). The color bar denotes the *t*-value. Error bars define the SEM. **(A–J)** Correlations among the altered DC and PSPD-related parameters (SAS and SDS). IOG, lateral occipital gyrus; CAL, calcarine fissure; PCL, paracentral gyrus; R, right; L, left; SAS, Self-Rating Anxiety Scale; SDS, Self-Rating Depression Scale.

**FIGURE 2:**
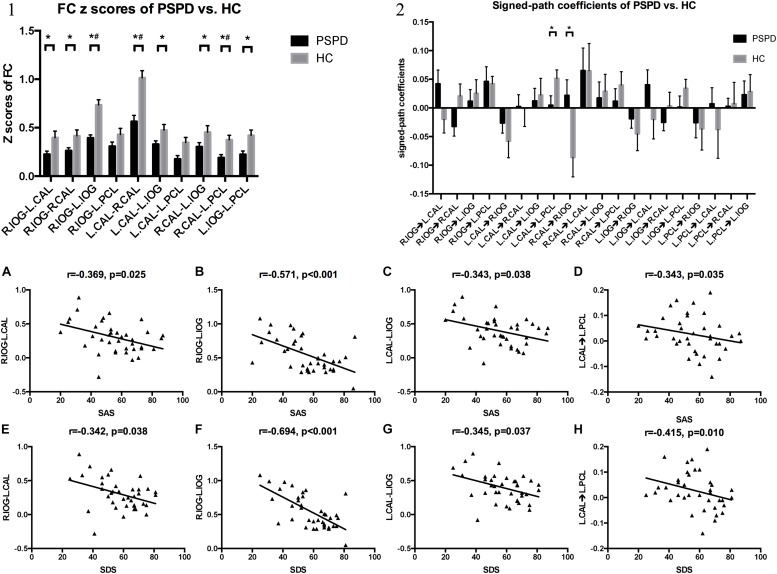
FC pattern and GCA pattern of inter-group comparisons, and the altered FC and signed-path coefficients significantly correlated with SAS and SDS. **(1)** Comparison of FC *z*-scores between the two groups. *P < 0.05, ^#^*P* < 0.05/10 (Bonferroni correction). **(2)** Comparison of signed-path coefficients between the two groups. *P < 0.05. Error bars define the SEM. **(A–C,E–G)** FC between right IOG and left CAL, right IOG and left IOG, left CAL and left IOG correlated with SAS and SDS. **(D,H)** Signed-path coefficients of the left CAL to the left PCL vs. SAS and SDS. IOG, lateral occipital gyrus; CAL, calcarine fissure; PCL, paracentral gyrus; R, right; L, left; SAS, Self-Rating Anxiety Scale; SDS, Self-Rating Depression Scale.

Compared to HCs, lower DC values were observed in the bilateral inferior occipital gyrus (IOG), bilateral calcarine fissure (CAL), and left paracentral lobule (PCL) of PSPD patients (GRF corrected: *P* < 0.001, *Z* > 3.09) (Table 2 and Figures 1-1,1-2).

**TABLE 2:** Brain regions with significant DC differences between the two groups.

Region	BA	MNI coordinates			Peak *t*-value	Cluster size (mm^3^)
		*x*	*y*	*z*		
R.lateral occipital gyrus	19	45	−69	−15	−4.489	27
L.calcarine fissure	17	−12	−90	−9	−4.532	35
R.calcarine fissure	18	12	−90	−1	−4.444	33
L.lateral occipital gyrus	19	−45	−81	−3	−5.139	35
L.paracentral gyrus	6	−3	−27	69	−4.471	37

*MNI, Montreal Neurological Institute; BA, Brodmann Area; R, right; L, left; (GRF corrected: *P* < 0.001, *Z* > 3.09).*

### Connectivity Analysis

Results obtained with the independent *t*-test indicated that FC values of right IOG–left CAL (*P* = 0.033), right IOG–right CAL (*P* = 0.036), right IOG–left IOG (*P* < 0.001), left CAL–right CAL (*P* < 0.001), left CAL–left IOG (*P* = 0.044), left CAL–left PCL (*P* = 0.012), right CAL–left PCL (*P* = 0.003), and left IOG–left PCL (*P* = 0.006) were lower in PSPD patients than in HCs. After multiple comparisons correction, the FC of the right IOG–left IOG, left CAL–right CAL, and right CAL–left PCL was lower in PSPD patients than in HCs (*P* < 0.05/10) (Figure 2-1).

In the GCA analyses, a negative causal effect from the left CAL to the left paracentral lobule (PCL) and a positive effect from the right CAL to the right IOG were observed in PSPD patients (*P* < 0.05) (Figure 2-2); however, the independent *t*-test results were non-significant after multiple comparisons correction (*P* > 0.05/20) (Figure 2-2).

### Correlation Analysis

A significant negative correlation was observed between DC values of the five above-mentioned brain regions (bilateral IOG, bilateral CAL, and left PCL) and SAS as well as SDS scores in all participants (*P* < 0.05) (Figures 1A–J).

FC values of right IOG–left CAL (*r* = -0.369, *P* = 0.025; *r* = −0.342, *P* = 0.038), right IOG–left IOG (*r* = −0.571, *P* < 0.001; *r* = −0.694, *P* < 0.001), and left CAL–left IOG (*r* = −0.343, *P* = 0.038; *r* = −0.345, *P* = 0.037) were negatively correlated with SAS and SDS scores (Figures 2A–C,E–G) in all participants. Signed-path coefficients from the left CAL to the left PCL were also negatively correlated with SAS and SDS scores (*r* = −0.343, *P* = 0.035; *r* = −0.415, *P* = 0.010; Figures 2D,H).

## Discussion

In this study we used a novel approach combining DC, FC, and GCA to investigate changes in PSPD patient multiple brain regions and the whole-brain functional network connectome. We found that PSPD patients showed abnormal network DC in multiple brain regions as well as abnormal FC between these regions as expected. The GCA analysis further revealed disordered connectivity from the left CAL to the left PCL and from the right CAL to the right IOG. Moreover, significant negative correlations were found between all abnormal network DC values, most abnormal FC values, signed-path coefficients from the left CAL to the left PCL, and SAS as well as SDS scores. These findings provide novel insight into the large-scale functional reorganization that occurs in the PSPD patient brain.

In humans, the IOG (BA19) is a visual association area that functions in feature extraction, shape recognition, attention, and multimodal integration, while the CAL harbors the primary visual cortex (V1) (Thiebaut de Schotten et al., 2014). We found that PSPD patients showed abnormal network DC in the bilateral IOG and bilateral CAL and lower FC values of bilateral CAL–right IOG, bilateral IOG, bilateral CAL, and left CAL–left IOG. Furthermore, GCA showed a disruption of direct connectivity from the right CAL to the right IOG, which is where abnormal FC was detected. Thus, PSPD patients exhibit altered brain functional hubs and connectivity in the occipital lobe, which is presumed to be is related to visual information processing (Sveinbjornsdottir and Duncan, 1993). However, the occipital cortex is connected to structures associated with inhibition of the descending pain pathway in the rats animal experiments (Reis et al., 2010). Several studies have demonstrated decreased signals in the occipital lobes of patients with chronic pain disorder by positron emission tomography, electroencephalogram, and rs-fMRI (Cauda et al., 2009; Karibe et al., 2010; Klug et al., 2011). Decreased ReHo in the occipital lobe was found to be negatively correlated with migraine duration (Zhao et al., 2013) and was observed in PSPD patients (Huang et al., 2016). GCA in current study showed the disruption of direct connectivity from the right CAL to the right IOG also showed less neural impulses in the occipital lobe of PSPD patients than of HC. We speculate that altered functional hubs and connectivity in this brain region contributes to the pathological impairment of sensation and pain-related emotion in PSPD. However, the relationship between abnormal activation of the occipital lobe and pain-related brain dysfunction remains to be elucidated.

The PCL controls motor and sensory innervation and plays an important role in somatosensation (Jellinger, 2008). Many studies have reported that the pain pathways of the human brain are widely distributed and encompass the somatosensory cortex, which is a sensory/motivational association area and involved in affective/discriminative aspects of pain (Xie et al., 2009; Seifert and Maihofner, 2011; Quintero, 2013). Neuroscientific research has indicated that the effects of expectancy on the subjective pain experience are paralleled by changes in somatosensory areas which are afferent nociceptive brain areas, and that this is partly mediated by descending pain modulatory circuits (Peters, 2015). A meta-analysis of PET, fMRI, EEG, and MEG studies provides clarity regarding the regions including primary and secondary somatosensory, insular, anterior cingulate and thalamus found active during an acute pain experience, investigating that these areas are the fundamental core network of human nociceptive processing (Apkarian et al., 2005). PSPD is a mental disorder with pain that is unrelated to somatic injury but can cause significant somatosensory and emotional disturbance (Egloff et al., 2014). Decreased ReHo were found in the bilateral primary somatosensory cortex of PSPD patients (Huang et al., 2016) and altered co-activation in the primary somatosensory cortex (Zhao et al., 2017). We found that PSPD patients had abnormal network DC in the PCL as well as lower FC values of bilateral CAL–left PCL and left IOG–left PCL. Our results suggest that pain-related networks are in fact highly interactive and disrupt intra-network FC within sensory and cognitive systems.

However, GCA showed disordered connectivity from the left CAL to the left PCL, which is where abnormal FC was detected. GCA result revealed more neural impulses or output information from visual cortex to somatosensory cortex but less from somatosensory cortex to visual cortex of PSPD patients than of HC. The combined effect might cause the lower FC between the left CAL and the left PCL. But inconsistent between the undirected and directed connectivity may result from the distinction of mathematical theories (Seth et al., 2015). So this phenomenon requires a further investigation.

The significant negative correlations observed between all abnormal network DC values, most abnormal FC values, signed-path coefficients from the left CAL to the left PCL, and SAS as well as SDS scores. Our results suggest that emotion perception could be affected by sensory/discriminative pain processing, consistent with the notion that various states of patients could influence on pain perception via sensory systems, such as anxiety, sadness, and depression (Schonenberg et al., 2014). We speculate that these characterization on large-scale functional networks and correlation could explain pain-related emotional disturbance in PSPD. Thus, psychological interventions that enhance emotional awareness may be beneficial for PSPD patients.

This study had a few limitations. Firstly, since the population size was relatively small, inter-group comparisons in GCA were non-significant after stringent multiple corrections, which affected not only the statistical power but also the interpretation of the results, especially the sample size, age distribution, and gender bias, which may be the reason that the relationship between DC, FC and clinical assessment within PSPD group wasn’t found. So the result needed to be replicated in larger samples. What’s more, one of the main limitation is the relatively long TR = 2 s which is sustantilly longer than typical inter-neuron delays, although most functional MRI study use this TR. EEG could offer millisecond-range temporal resolution which is not possible with MRI (Britz et al., 2010). EEG is a widely utilized technique to measure oscillatory activity in different frequency bands originated by neuronal assemblies, and to link these oscillations with specific behavioral states (Britz et al., 2010; Xu et al., 2010, 2015). There is accumulating evidence that the spatial distribution of scale-free properties was compatible between EEG and fMRI (Lei et al., 2011; Xu et al., 2014). We are also considering the application of using simultaneous EEG-fMRI to get a more complete characterization of scale-free organization of brain in PSPD.

## Conclusion

We took the novel approach of using a combined DC, FC, and GCA approach to investigate the changes in the PSPD patients brain functional hubs and their connectivity, and found altered large-scale functional networks involved occipital lobe and somatosensory cortices, which may mirror the underlying neural mechanism of the dysfunction of sensation and pain-related emotional disturbances in PSPD patients.

## Author Contributions

QL conceived and executed the project and wrote the first draft of the manuscript. X-cZ performed the MRI data analysis. X-MJ contributed to the participant recruitment and clinical data acquisition. Z-hZ and X-fH contributed to the project conception, manuscript review, and critique.

## Conflict of Interest Statement

The authors declare that the research was conducted in the absence of any commercial or financial relationships that could be construed as a potential conflict of interest.

## Footnotes

1 http://www.restfmri.net

2 http://www.fil.ion.ucl.ac.uk/spm

3 http://restfmri.net/forum/index.php
